# THE NATIONAL STUDY OF DAILY EXPERIENCES: CHARTING QUOTIDIAN LIFE OF ACROSS ADULTHOOD

**DOI:** 10.1093/geroni/igae098.1858

**Published:** 2024-12-31

**Authors:** David Almeida

**Affiliations:** Pennsylvania State University, University Park, Pennsylvania, United States

## Abstract

This presentation delves into the intricate tapestry of daily experiences across the adult lifespan, drawing insights from the MIDUS daily project of a US national sample of 3,509 individuals. Through analysis of over 42,000 daily telephone interviews collected across 20 years, we investigate the nuances of quotidian life, uncovering patterns, and determinants that shape individuals’ daily routines and emotional landscapes. Leveraging a diverse sample spanning different age groups, socio-economic backgrounds, and geographic locations, we illuminate the multifaceted nature of daily experiences. Findings reveal dynamic trajectories of well-being, stress, and activities, shedding light on how these dimensions fluctuate with age and life circumstances. Furthermore, we explore the influence of contextual factors such as work, family, and social interactions on daily experiences, unraveling their complex interplay with individual characteristics. This research contributes to a deeper understanding of the lived realities of adults, offering implications for enhancing daily well-being and quality of life.

